# Development of a Kinetic Model for the Redox Reactions of Co_2.4_Ni_0.6_O_4_ and SiO_2_/Co_2.4_Ni_0.6_O_4_ Oxides for Thermochemical Energy Storage

**DOI:** 10.3390/ma15103695

**Published:** 2022-05-21

**Authors:** Yasmina Portilla-Nieto, Daniel Bielsa, Jean-Luc Dauvergne, Marta Hernaiz, Estibaliz Aranzabe, Stefania Doppiu, Elena Palomo del Barrio

**Affiliations:** 1 Centre for Cooperative Research on Alternative Energies (CIC energiGUNE), Basque Research and Technology Alliance (BRTA), Alava Technology Park, Albert Einstein 48, 01510 Vitoria-Gasteiz, Spain; dbielsa@cicenergigune.com (D.B.); jldauvergne@cicenergigune.com (J.-L.D.); sdoppiu@cicenergigune.com (S.D.); e.palomo@cicenergigune.com (E.P.d.B.); 2 Applied Physics II Department, Faculty of Science and Technology, University of the Basque Country (UPV/EHU), 48080 Bilbao, Spain; 3 TEKNIKER, Polo Tecnológico de Eibar, C/Iñaki Goenaga, 5, 20600 Eibar, Spain; marta.hernaiz@tekniker.es (M.H.); estibaliz.aranzabe@tekniker.es (E.A.); 4 IKERBASQUE Basque Foundation for Science, Plaza Euskadi 5, 48009 Bilbao, Spain

**Keywords:** thermochemical heat storage, gas-solid reaction, reduction/oxidation reaction, cobalt–nickel oxide, kinetic models

## Abstract

One of the possible solutions for the transition of the actual energetic model is the use of thermal energy storage technologies. Among them, thermochemical energy storage based on redox reactions involving metal oxides is very promising due to its high energy density. This paper deals with the development of the kinetic study based on data extracted from the thermogravimetric analysis of a cobalt-nickel mixed oxide (Co_2.4_Ni_0.6_O_4_) without and with the addition of SiO_2_ particles to improve the cyclability. The results show that in the reduction reaction the activation energy is not affected by the addition of SiO_2_ particles while in the oxidation reaction an increase in the activation energy is observed. The theoretical models fitting with the experimental data are different for each material in the reduction reaction. The mixed oxide is controlled by a nucleation and growth mechanism for conversion ratios higher than 0.5, while the added material is controlled by diffusion mechanisms. In the oxidation reaction, the two materials are controlled by a nucleation and growth mechanism for conversion ratios higher than 0.5.

## 1. Introduction

Excessive energy use on a global scale and the associated problems have meant a focused interest in the field of renewable energy. Among these, Concentrated Solar Power (CSP) is of great interest due to the wide availability of solar energy, its cost efficiency and ease of hybridization [1]. The main drawback of this technology is its intermittency, making storage systems necessary to guarantee the availability of energy. This problem can be solved by using large-scale and inexpensive thermal energy storage (TES) systems. Moreover, the combination of CSP plants with TES contributes to making them economically viable [2].

Among the TES technologies, there are two more developed, which are sensible heat storage (SHS), where the energy is stored by increasing the temperature of the storage material and released when decreasing the temperature, and latent heat storage (LHS), where the energy storage is carried out through phase changes of the storage material [3,4,5]. Currently, the focus is on the development of the less studied but most promising TES technology, thermochemical storage (TcES), based on the use of high enthalpic reversible chemical reactions to store or release energy. This technology theoretically provides a much higher energy storage density than other TES technologies [6,7]. During the on-sun hours, the endothermic chemical reaction is used to store heat and during the off-sun hours, the reverse exothermic reaction is used to release heat.

Previous research works have selected redox reactions involving metal oxides as suitable candidates for high-temperature TES applications. Therefore, redox systems need suitable materials which should fulfill requirements such as complete reaction reversibility, suitable reaction temperature, high storage density, high reaction enthalpy, no toxicity and good thermal stability during cycling in the operating temperature range [8,9,10].

Different research groups have investigated different materials which can be suitable for thermochemical energy storage at high temperatures. The most favored resulting materials are metal oxides due to their high reaction enthalpies, high operating temperature ranges and utilization of air at the same time as a reactant and a heat transfer fluid [11,12,13].

The redox reactions can be presented as [14]:

Reduction reaction: MxOy+z→MxOy+z2O2

Oxidation reaction: MxOy+z2O2→MxOy+z

where *M* is a metal.

The most studied metal oxides for thermochemical applications have been cobalt oxide (Co_3_O_4_/CoO) and manganese oxide (Mn_2_O_3_/ Mn_3_O_4_) [8,11,15,16], but their reaction temperatures (higher than 850 °C) being too high is considered as the main barrier for their final application in technologies such as CSP or industrial waste heat recovery.

The literature shows that the development of mixed oxides is a correct alternative to tuning the reaction temperatures. The main problem is that, in most cases, the result is an increase in the reaction temperature instead of a decrease [17,18]. Sometimes, the development of mixed oxides can improve long-term cyclability. Another alternative for improving the cyclability without harming the reaction temperatures is the addition of nanoparticles to the mixed oxides [19,20].

Co_3_O_4_ has a spinel structure with the general formula of AB_2_O_4_ where A, B = Co, Zn, Ni, Fe, Cu, Mn, etc. A and B are divalent and trivalent metal cations, respectively. The nature of the cations incorporated into the structure affects the distribution of the cations between the two sites. In common spinel structures, the divalent A and trivalent B cations occupy the tetrahedral and octahedral sites, respectively [21,22,23].

Previous research works have demonstrated that developing mixed oxides based on Co–Ni allows for tuning the reaction temperatures depending on the amount of nickel in the cobalt oxide host structure. The most promising formulation was Co_2.4_Ni_0.6_O_4_, obtaining reaction temperatures of around 700 °C during 100 thermal cycles in TGA [24]. The activity loss shown during the cycling process was studied by adding SiO_2_ particles in the Co_2.4_Ni_0.6_O_4_, showing no significant differences in the behavior of the material in a limited number of cycles, contrary, the 20-year predictions (4000 cycles) anticipate a notably better behavior of the mixed oxide with the addition than the one without it. This work is under review.

Several works focusing on the kinetic studies of pure cobalt oxide were published [25,26,27,28]. Some studies have found that the reduction and oxidation reactions of Co_3_O_4_/CoO are controlled by the heat transfer and diffusion mechanism, respectively [18]. Other studies have analyzed the effect of different additives, such as Al_2_O_3_ and Y_2_O_3_, in the cobalt oxide, demonstrating that the Co_3_O_4_–Al_2_O_3_ desorbs more oxygen than the Co_3_O_4_–Y_2_O_3_ in the same conditions, and the activation energy of Co_3_O_4_–Al_2_O_3_ and Co_3_O_4_–Y_2_O_3_ changes proportionally to the conversion fraction (α). These effects were attributed to the different ionic radii presented by the aluminum oxide and the yttrium oxide, the ability to create new compounds with different decomposition temperatures and their effect on the sintering of cobalt oxide [29].

In this work we studied, for the first time, the kinetic mechanisms of Co_2.4_Ni_0.6_O_4_ and of the 0.5% SiO_2_/Co_2.4_Ni_0.6_O_4_ with the objective of studying the effects of Ni in the cobalt oxide structure and to obtain a comparison of the kinetic parameters (activation energy (Ea), preexponential factor (A) and reaction model (f(α))) for the nickel cobaltite and the added nickel cobaltite.

The redox reaction of the metal oxides studied in this research is:

Co3−xNixO4↔3Co1−xNixO+12O2

and the maximum conversion reached for the Co_2.4_Ni_0.6_O_4_ material is with a mass gain/loss of 6.65%.

## 2. Materials and Methods

The synthesis of the mixed oxides was performed with the materials: nickel nitrate hexahydrate extra-pure from Scharlab (Barcelona, Spain, EU), cobalt nitrate hexahydrate from Fisher Scientific (Pittsburgh, PA, USA) with a purity of 98^+^%, citric acid from Fisher Scientific with a purity of 99^+^% and ethylene glycol anhydrous from Sigma-Aldrich (Saint Louis, MO, USA) with a purity of 99.8%.

For the synthesis of the SiO_2_ particles, the used materials were: tetraethyl orthosilicate (TEOS) from ACROS Organics with a purity of 98%, ethanol absolute form Scharlau, distilled water and an ammonia solution from EMSURE with a purity of 28–30%.

The synthesis of Co–Ni mixed oxides was carried out following the sol–gel Pechini Route. For this purpose, stoichiometric quantities of Co(NO_3_)_2_·6H_2_O and Ni(NO_3_)_2_·6H_2_O nitrates were used. First of all, the primary precursors were dissolved in ethylene glycol under magnetic stirring to obtain a homogeneous solution. Subsequently, citric acid was added to the previous mixture under vigorous stirring for 1 h. Once the solution mixture was well mixed, it was dried overnight at 180 °C. The resulting powders were ground in an agate mortar to increase their homogeneity and then calcined in air at 400 °C for 10 h.

The Stober route was used for the synthesis of SiO_2_ particles. Appropriate quantities of tetraethyl orthosilicate, ammonia, distilled water and ethanol were used to produce SiO_2_ particles. Firstly, two solutions were prepared under magnetic stirring: (i) 2/3 of ethanol with ammonia and distilled water (solution A); and (ii) TEOS with 1/3 of ethanol (solution B). Solution A was maintained at ambient temperature under magnetic stirring. Subsequently, solution B was added to solution A and was kept there for 25 hours for aging. Once the particles were formed, the remaining solvent of the mixture was removed in a rotary evaporator.

The addition of a 0.5 wt. % of silica particles in the cobalt–nickel mixed oxide was carried out using an ultrasound tip using an amplitude of 50%, cooling the mixture to avoid agglomerates. The mixture was dissolved in 100 mL of ethanol and dispersed for 1 min.

The equipment used during the research was:

Thermogravimetric analysis (TGA). The samples were analyzed in a TGA/DSC 1 from Mettler Toledo, using a sensor type DSC HSS2, a furnace LF heating until 1100 °C, equipped with a sample robot standard. The gas controller is the type GC 200. The amount of material analyzed each time was 20 mg, and the temperature range was from 600 °C to 910 °C, using different heating/cooling ramps depending on the point of the model development.

The validation model was constructed using the Octave software.

## 3. Results

### 3.1. Kinetic Analysis

The kinetic models are usually determined by the intrinsic mass gained/lost regarding the oxygen absorption/desorption of metal oxides. For this purpose, the conversion ratio (*α*) is plotted against time (*t*).

The conversion ratio (*α*) is defined in Equation (1) [30]:

(1) $$\alpha =\frac{m_{0}-m_{t}}{m_{0}-m_{f}}$$

where *m*_0_ is the initial mass, *m_t_* is the mass at time *t* and *m_f_* is the final mass.

The parameters to be obtained for the development of a kinetic model are mainly: activation energy (*Ea*), preexponential factor (*A*) and reaction model (*f*(*α*)) [31].

The reaction rate can be described by an Arrhenius type law [31]:

(2) $$\frac{d\alpha }{dt}=k\left(T\right)\cdot f\left(\alpha \right)$$

(3) $$k\left(T\right)=Ae^{-\frac{E_{a}}{RT}}$$

where *R* is the universal gas constant and *T* the temperature.

Combining Equations (2) and (3), Equation (4) is obtained:

(4) $$\frac{d\alpha }{dt}=Ae^{-\frac{E_{a}}{RT}}\cdot f\left(\alpha \right)$$

The solution of Equation (4), and thus of the kinetic model, can be carried out using different analytical methods such as model-fitting methods, generalized kinetic models and isoconversional methods, the most used ones for metal oxides in TcES [32].

The most common isoconversional method is the Friedman method [33] and is based on the calculation of the activation energy (*Ea*) without knowing the kinetic model (*f*(*α*)) by assuming that the reaction rate is a function of the temperature for an extent of the conversion (*α*). Equation (4) can be written in its logarithmic form as:

(5) $$ln\left(\frac{d\alpha }{dt}\right)=ln\left(Af\left(\alpha \right)\right)-\frac{E_{a}}{RT}$$

A plot of the left term of Equation (5) vs. *1/T* allows for obtaining *Ea* from the slope of the curve at different heating/cooling rates. Previous research works say that this method gives more accurate values of activation energy than the Ozawa method [34].

Once the activation energy is calculated, the next step is to obtain the reaction model *f*(*α*) by using the master plots method. For this purpose, the value of the activation energy obtained and the experiments carried out with the TGA, at different heating/cooling rates, need to be used. The determination of *f*(*α*) is based on the comparison of an experimental master plot with the theoretical ones listed in Table 1.

To obtain the experimental master plot is necessary to express the kinetic rate equation (Equation (4)) at infinite temperature by introducing the generalized time *θ* as [35,36,37]:

(6) $$\theta =\int_{0}^{t}exp\left(-\frac{E_{a}}{RT}\right)dt$$

where *θ* denotes the reaction time taken to attain a particular α at an infinite temperature [36]. The differentiation of Equation (6) gives [35,36,37]:

(7) $$\frac{d\theta }{dt}=exp\left(-\frac{E_{a}}{RT}\right)$$

By combining Equations (4) and (7), the next equation is obtained [35,36,37]:

(8) $$\frac{d\alpha }{d\theta }=Af\left(\alpha \right)$$

Or:

(9) $$\frac{d\alpha }{d\theta }=\frac{d\alpha }{dt}exp\left(\frac{E_{a}}{RT}\right)$$

Equation (8) can be derived using a reference point at *α* = 0.5 obtaining [36].

(10) $$\frac{d\alpha /d\theta }{{\left(d\alpha /d\theta \right)}_{\alpha =0.5}}=\frac{f\left(\alpha \right)}{f\left(0.5\right)}$$

Combining Equations (9) and (10), the final equation for the obtention of the experimental master plot is reached [36]:

(11) $$\frac{d\alpha /d\theta }{{\left(d\alpha /d\theta \right)}_{\alpha =0.5}}=\frac{d\alpha /dt}{{\left(d\alpha /dt\right)}_{\alpha =0.5}}\frac{exp\left(E_{a}/RT\right)}{exp\left(E_{a}/RT_{0.5}\right)}$$

The experimental master plot is obtained by representing the right side vs. conversion.

If the experimental master plot does not fit completely with the theoretical models listed in Table 1, the Sestak–Berggren (SB) model [38,39] can be used, which is a mathematical description of most of the possible solid-state reaction mechanisms. In general, it does not provide information about the mechanisms involved in the reaction but allows us to model almost any reaction process [38]. This empirical model can be calculated as:

(12) $$f\left(\alpha \right)=\alpha^{m}{\left(1-\alpha \right)}^{n}{\left[-ln\left(1-\alpha \right)\right]}^{p}$$

where *m*, *n* and *p* are kinetic exponents that fit with the experimental data.

Once *Ea* and *f*(*α*) are determined, the calculation of the preexponential factor *A* can be directly carried out by applying Equation (5).

### 3.2. Results and Discussion

Once the morphological and structural characterization was completed for the mixed oxide Co_2.4_Ni_0.6_O_4_ [24] and for the Co_2.4_Ni_0.6_O_4_ with the addition of 0.5% SiO_2_ particles (under review), a kinetic study of the two materials was carried out. In the previous research work, the correct synthesis of the mixed-phase was determined by a deep structural (by XRD), and morphological (by SEM) analysis and an evaluation of the degree of reversibility (TGA measurements) [24].

The interactions between the SiO_2_ particles and the Co_2.4_Ni_0.6_O_4_ was studied by in situ XRD demonstrating the absence of interactions between the two materials. The XRD patterns showed no SiO_2_ phases as observed in other research works [40] and the unit cell parameter shows no entry of SiO_2_ into the nickel cobaltite structure. The morphology of the mixed oxide with the addition of SiO_2_ was assessed by means of SEM, showing larger particles than in the case of the Co_2.4_Ni_0.6_O_4_ but less compacted, allowing the oxygen to flow through the material. The cyclability was characterized by TGA, showing that the effect of the particles is not macroscopic in short cycles but helps to keep the reduction temperatures lower than in the case of nickel cobaltite. A prediction of the material based on the experimental results showed a clear effect on long-term cycling, keeping conversion levels higher than in the case of the Co_2.4_Ni_0.6_O_4_ without any addition.

**Figure 1:**
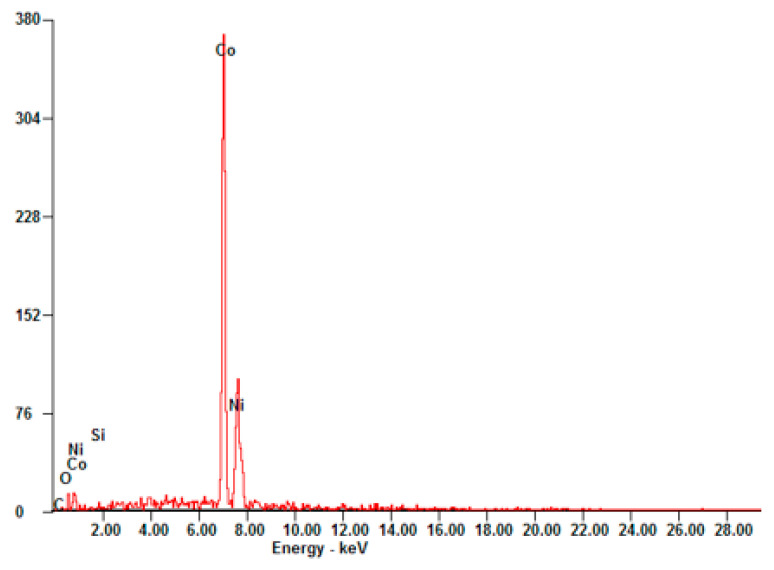
EDX result for the 0.5% SiO_2_/Co_2.4_Ni_0.6_O_4_ material.

It should be noted that all the materials used for the development of the kinetic models were examined by EDX to guarantee the desired stoichiometry. The theoretical Co:Ni ratio should be 80:20 in the case of Co_2.4_Ni_0.6_O_4_ and the experimental value obtained was 78.55:21.45, in the range of the experimental error. The results are depicted in Figure 1 and listed in Table 2.

The materials used underwent a previous thermal treatment based on 50 cycles in TGA to analyze the definitive kinetics of the material once it had stabilized in terms of grain size, morphology, etc.

#### 3.2.1. Reduction Reaction Rate

For the obtainment of the kinetic parameters of the reduction reaction rate of Co_2.4_Ni_0.6_O_4_ and 0.5% SiO_2_/Co_2.4_Ni_0.6_O_4_ and to study the effect of the silica particles in the kinetics of the mixed oxide, different heating/cooling ramped experiments were performed in a TGA. The ramps used were: 5, 10, 15 and 20 °C/min. All the experiments were performed under an N_2_ atmosphere to avoid the influence of the oxygen pressure (pO_2_) in the analysis.

**Figure 2:**
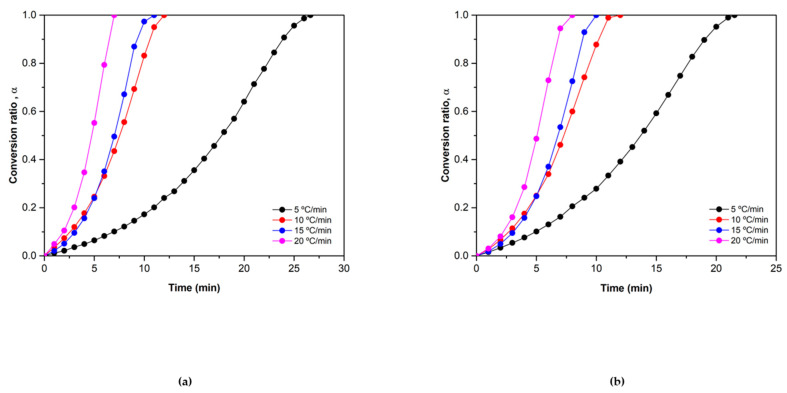
α vs. time plots of the reduction reaction of (**a**) Co_2.4_Ni_0.6_O_4_ and (**b**) 0.5% SiO_2_/Co_2.4_Ni_0.6_O_4_.

Figure 2 shows that the mixed oxide with the addition of particles reaches the total conversions faster than the mixed oxide without the addition in most of the heating ramps. The bigger difference is shown in the 5 °C/min heating ramp, in which the pure material takes 27 min to reach the total conversion and the added one only 22 min. In the initial part of the experiment, it can be observed that the conversion ratio of the 10 °C/min experiment is slightly higher than the one of the 15 °C/min experiment. This effect is attributed to the experimental error of the instrument as the conversion values in low conversion ratios are similar.

The Friedman method was used for obtaining the activation energy. The data taken from TGA were treated following Equation (5). Plotting the left term of Equation (5) vs. the inverse of the temperature (1000/*T*) at different extents of reaction (*α*) and heating rates, allows us to determine the *Ea* of the material by measuring the slope of the curves.

It should be noted that for the calculation of the activation energies, the trendlines with R^2^ lower than 0.97 have not been considered. In this case, the two trendlines obtained in the conversion ratio of 0.2 were discarded as their R^2^ value was 0.96 for Co_2.4_Ni_0.6_O_4_ and 0.71 for 0.5% SiO_2_/Co_2.4_Ni_0.6_O_4_.

**Figure 3:**
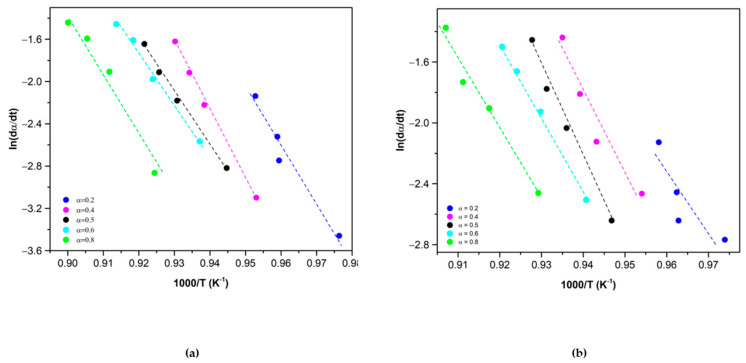
Friedman plot for the reduction of (**a**) Co_2.4_Ni_0.6_O_4_ and (**b**) 0.5% SiO_2_/Co_2.4_Ni_0.6_O_4_ for different conversion ratios.

The values of the average activation energy obtained from the slope of the curves of Figure 3 are: 450 ± 47 kJ/mol in the case of the Co_2.4_Ni_0.6_O_4_ and 449 ± 32 kJ/mol for the added 0.5% SiO_2_/Co_2.4_Ni_0.6_O_4_. Previous research works have given very different *Ea* values for the pure Co_3_O_4_. Muroyama et al. report a value of 247 kJ/mol [25], while Wong et al. report 960 kJ/mol [26] and Hasanvard et al. report 158.99 kJ/mol as the maximum value for a determined α [29]. However, some authors state that the activation energy can be highly dependent on the experimental conditions, sample preparation and determination of the reaction mechanism [25]. Previous research works have determined the activation energies of some doped Co_3_O_4_ materials too. Wong et al. reported an activation energy of 731 kJ/mol in the case of the 5% Al_2_O_3_/Co_3_O_4_ [26] while Hasanvard et al. reported 238 kJ/mol [29] as the maximum activation energy for the same material.

Comparing the activation energy obtained in this work for Co_2.4_Ni_0.6_O_4_ and the values obtained in other research works for Co_3_O_4_ and doped Co_3_O_4_, the results are in the interval of activation energies calculated by the other authors. Taking into account that the reduction temperature of the mixed nickel cobaltite is lower than the reduction temperature of the pure cobalt oxide, the activation energy should be lower too.

**Figure 4:**
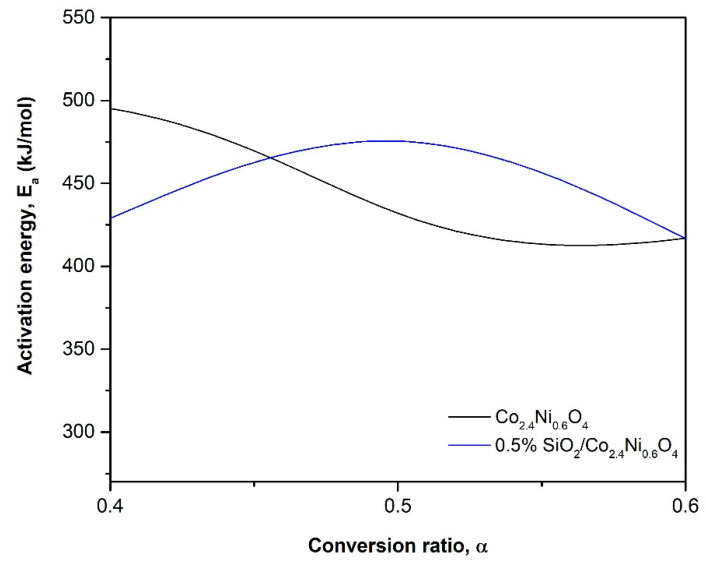
Evolution of the activation energy (*Ea*) with the reduction conversion ratios (*α*).

Regarding the comparison between Co_2.4_Ni_0.6_O_4_ and 0.5% SiO_2_/Co_2.4_Ni_0.6_O_4_, the same *Ea* was obtained and the deviation was quite similar for the two materials, as can be seen in Figure 4.

**Figure 5:**
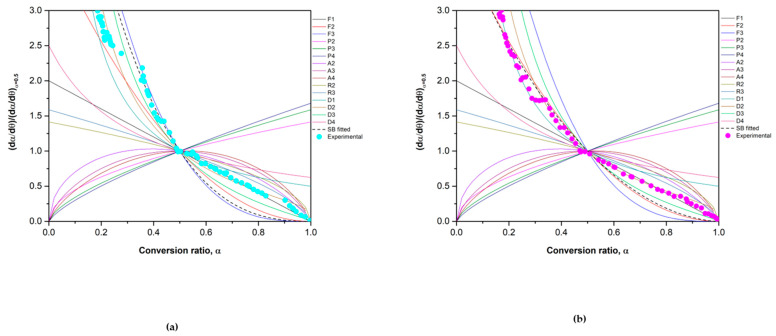
Theoretical master plots, SB fitted function and experimental results of (**a**) Co_2.4_Ni_0.6_O_4_ and (**b**) 0.5% SiO_2_/Co_2.4_Ni_0.6_O_4_ using a heating ramp of 20 °C/min.

Once the average activation energy was determined, the next step was to determine the reaction mechanism using the master plot method. Introducing the activation energy obtained in Equation (11) allows us to obtain an experimental master plot and makes its comparison with the theoretical models listed in Table 1 possible. The results are depicted in Figure 5.

The Co_2.4_Ni_0.6_O_4_ material shows a different behavior depending on the conversion ratio. When α is lower than 0.5, the experimental data fit partially with the model F3 and for an α higher than 0.5, the model F1 correctly describes the behavior of Co_2.4_Ni_0.6_O_4_. These models describe instantaneous nucleation and unidimensional growth [36]. The SB model was used to define the model of the whole experiment but the only successful fitting corresponded to the *α* between 0.3 and 0.5, by using the exponents *m* = 2, *n* = 1.7 and *p* = −2.1, obtaining the SB equation:

(13) $$f\left(\alpha \right)=\alpha^{2}{\left(1-\alpha \right)}^{1.7}{\left[-ln\left(1-\alpha \right)\right]}^{-2.1}$$

Regarding the 0.5% SiO_2_/Co_2.4_Ni_0.6_O_4_ material, when α is lower than 0.2 the best theoretical model is D1, but for *α* values between 0.3 and 1, the experimental data fit well with a D2 model. In any case, these two theoretical models belong to the group of diffusion models, which are quite common in this type of material. The SB model was used to obtain an accurate model of the whole reaction extent. The results show that the fitting is correct when *α* is lower than 0.5 and the exponents are: *m* = 0.81, *n* = 1.45, and *p* = −0.87, obtaining the SB equation:

(14) $$f\left(\alpha \right)=\alpha^{0.81}{\left(1-\alpha \right)}^{1.45}{\left[-ln\left(1-\alpha \right)\right]}^{-0.87}$$

The fitting problems for the two materials are due to the oscillation that the *Ea* has in the whole range of conversions, as is depicted in Figure 4. If the activation energy is roughly constant in all the conversion ranges and there are no shoulders in the reaction rate curve, the process can be fitted by a single-step model. If not, depending on the conversion, different models describe the reaction at different stages [31].

Once the *f*(*α*) was defined, the “kinetic triplet” could be completed by calculating the preexponential factor (*A*) from the intercept of Equation (5). In the case of nickel cobaltite, the *A* is 9.65·1019 min^−1^, while in nickel cobaltite with an addition, the *A* is 6.37·1019 min^−1^.

To sum up, the kinetic triplets obtained for the Co_2.4_Ni_0.6_O_4_ and 0.5% SiO_2_/Co_2.4_Ni_0.6_O_4_ materials are listed in Table 3.

Thus, the equation describing the reduction reaction for the Co_2.4_Ni_0.6_O_4_ is:

(15) rred=dαreddt=9.65·1019min−1e(−449.82kJmolRT)αred2(1−αred)1.7[−ln(1−αred)]−2.1

And the resulting equation describing the model of the reduction reaction for the added 0.5% SiO_2_/Co_2.4_Ni_0.6_O_4_ material is:

(16) rred=dαreddt=6.37·1019min−1e(−449.22kJmolRT)αred0.81(1−αred)1.45[−ln(1−αred)]−0.87

#### 3.2.2. Oxidation Reaction Rate

For the obtention of the oxidation kinetic triplet for the Co_2.4_Ni_0.6_O_4_ and 0.5% SiO_2_/Co_2.4_Ni_0.6_O_4_ materials, isothermal programs ranging from 600 °C to 800 °C were performed under an O_2_ atmosphere (*p*O_2_ = 1). To prevent any oxidation before doing the experiments, all the materials were firstly reduced under N_2_ conditions and when the temperature of the experiment was stabilized, the atmosphere was changed to O_2_ for completing the oxidation. It should be noted that in the very beginning (first minute) of the experiment, the kinetics could be slightly influenced by the gas atmosphere change from N_2_ to O_2_.

**Figure 6:**
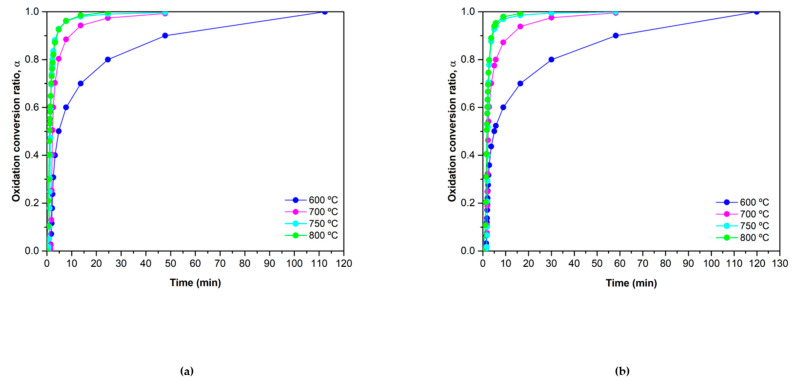
α vs. time plots of the oxidation reaction at *p*O_2_ = 1 of (**a**) Co_2.4_Ni_0.6_O_4_ and (**b**) 0.5% SiO_2_/Co_2.4_Ni_0.6_O_4_.

The conversion ratio as a function of the time is presented in Figure 6 for each isothermal experiment.

When the model design is performed by performing isothermal experiments instead of dynamic ones, it is not possible to follow the protocol previously followed for the obtention of the model of the reduction reaction, as in this case the temperature is constant and the activation energy cannot be calculated from Equation (5) directly. In this case, it is necessary to apply the master plots method without the *Ea* [31].

The theoretical master plot models are listed in Table 1. The obtention of the experimental master plots was carried out by using Equation (11) setting the term related to the temperatures and the activation energy as a constant:

(17) $$\frac{d\alpha /d\theta }{{\left(d\alpha /d\theta \right)}_{\alpha =0.5}}=\frac{d\alpha /dt}{{\left(d\alpha /dt\right)}_{\alpha =0.5}}$$

**Figure 7:**
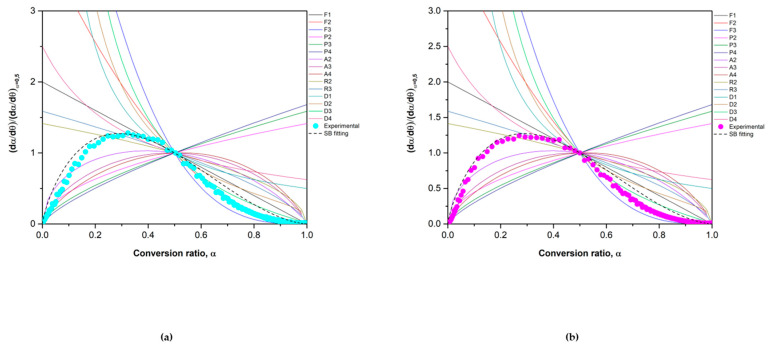
Comparison between theoretical models, experimental results and SB fitting of the experimental results for (**a**) Co_2.4_Ni_0.6_O_4_ and (**b**) 0.5% SiO_2_/Co_2.4_Ni_0.6_O_4_.

The results are shown below in Figure 7:

The experimental data obtained for Co_2.4_Ni_0.6_O_4_ do not present exactly the same tendency as any theoretical model listed in Table 1. In this case, both the material without addition and the material with the addition of silica shows a very similar experimental trend. Both materials fit well with the F2 model for *α* above 0.5. The F2 theoretical model corresponds to a reaction of second-order, where random nucleation is followed by an instantaneous growth of nuclei [32]. In order to obtain a model that fits well with the whole experimental data, the SB equation was used [38]. The behavior of Co_2.4_Ni_0.6_O_4_ and 0.5% SiO_2_/Co_2.4_Ni_0.6_O_4_ is so similar that the resulting exponents in the fit were the same for both materials. The exponents obtained were *m* = 2.8, *n* = 1 and *p* = −2.01. The SB equation obtained for both materials was:

(18) $$f\left(\alpha \right)=\alpha^{2.8}{\left(1-\alpha \right)}^{1}{\left[-ln\left(1-\alpha \right)\right]}^{-2.01}$$

In the case of the Co_2.4_Ni_0.6_O_4_ (Figure 7A), the SB equation fits with the experimental data in the *α* range from 0.25 to 0.5. In the rest of the range, the empirical method is close to the experimental results but does not fit completely well. This is a detriment when calculating the activation energies of the materials since the calculated *f*(*α*) has to be used. In the case of the added 0.5% SiO_2_/Co_2.4_Ni_0.6_O_4_ material (Figure 7B), the empirical calculation fits well with the experimental data from *α* = 0 to *α* = 0.5. The result of the rest of the range is quite similar but it does not fit perfectly. However, the oxidation reaction was further modeled using the values obtained for *f*(*α*), as it was the model that better described the whole experiment.

So then, for the obtention of an average value of *Ea*, the isoconversional method of Friedman was applied [33]. The isothermal experiments used for the calculation of the reaction model under the O_2_ atmosphere were used for the obtention of the Friedman plots.

**Figure 8:**
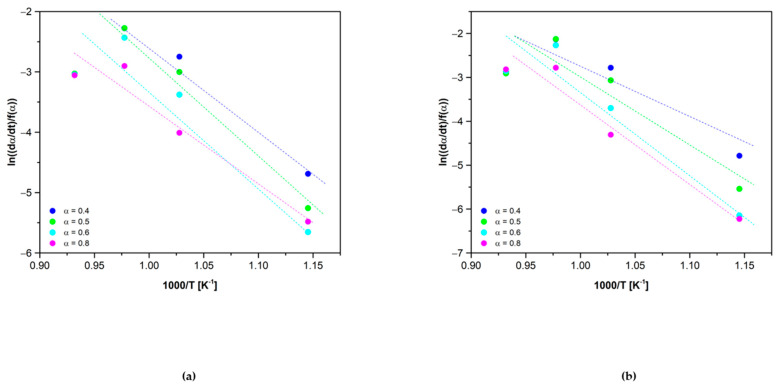
Friedman plots of (**a**) Co_2.4_Ni_0.6_O_4_ and (**b**) 0.5% SiO_2_/Co_2.4_Ni_0.6_O_4_ at different α.

It is worth mentioning that the trendlines observed in Figure 8 were obtained using four points, each one obtained from a different temperature isothermal analysis. In some cases, in the figure, one of the points overlapped for different conversion ratios.

The average oxidation activation energy obtained for the Co_2.4_Ni_0.6_O_4_ material was 100 ± 22 kJ/mol. The results obtained in this research work are quite similar to the ones reported in the bibliography regarding the Co_3_O_4_. Muroyama et al. report an oxidation activation energy of 58.07 ± 0.26 kJ/mol [25]. In the case of Reti et al., the oxidation activation energy obtained was 60.19 kJ/mol [41] and in the work of Tomlinson and Esterlow, the value was 80 ± 15 kJ/mol [42]. Regarding the doped Co_3_O_4_, Wong et al. reported an oxidation activation energy of 165 kJ/mol for the material 5%Al_2_O_3_/Co_3_O_4_ [26]. The result obtained in this research work is in between the values previously reported for pure and doped cobalt oxides. Regarding the 0.5% SiO_2_/Co_2.4_Ni_0.6_O_4_ material, an average oxidation *Ea* of 124 ± 36 kJ/mol was obtained. It is worth mentioning that the method selected for the obtention of the kinetic model can lead to different values of activation energy. The model *f*(*α*) selected previously can affect the results obtained too.

**Figure 9:**
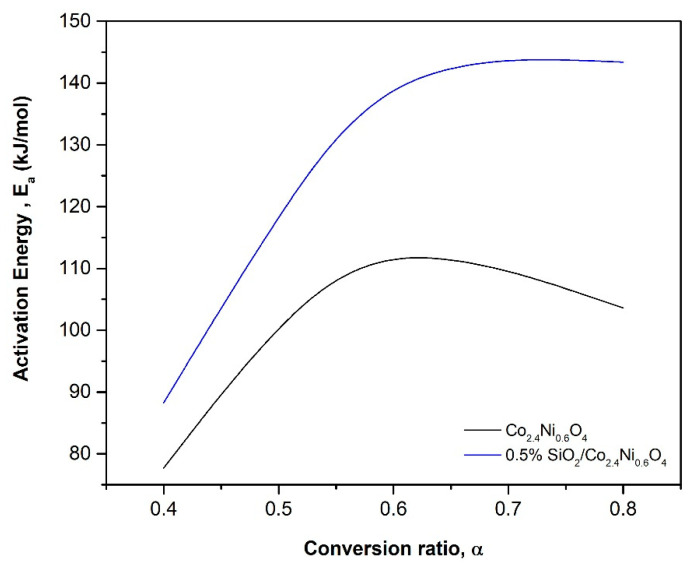
Evolution of the activation energy (*Ea*) with the oxidation ratio (*α*).

As with what happened in the reduction reaction, the activation energy of the oxidation reaction changes for each conversion ratio, as it can be observed in Figure 9. In the oxidation reaction, the two materials fitted well with the theoretical model F2 but only for a concrete conversion range, so the change of activation energy fits well with the result obtained for the model.

The preexponential factor (*A*) was calculated from the order at the origin of the Friedman plots shown in Figure 8 and the results obtained were 7·103 min^−1^ for the Co_2.4_Ni_0.6_O_4_ and 7.81·105 min^−1^ for the 0.5% SiO_2_/Co_2.4_Ni_0.6_O_4_.

To sum up, the “kinetic triplet” obtained for the oxidation reaction of the Co_2.4_Ni_0.6_O_4_ and SiO_2_/Co_2.4_Ni_0.6_O_4_ materials are listed in Table 4:

Thus, the equation describing the oxidation reaction for the Co_2.4_Ni_0.6_O_4_ is:

(19) rox=dαoxdt=7·103min−1e(−100kJmolRT)αox2.8(1−αox)1[−ln(1−αox)]−2.01

And the resulting equation describing the model of the oxidation reaction for the added 0.5% SiO_2_/ Co_2.4_Ni_0.6_O_4_ material is:

(20) rox=dαoxdt=7.81·105min−1e(−124kJmolRT)αox2.8(1−αox)1[−ln(1−αox)]−2.01

**Figure 10:**
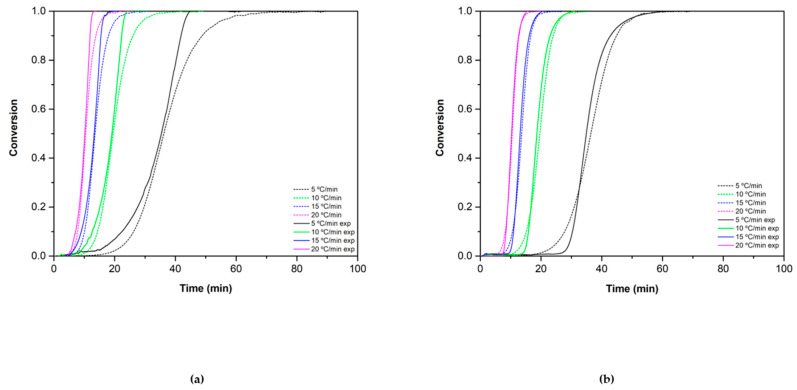
Reduction conversion validation for (**a**) Co_2.4_Ni_0.6_O_4_ and (**b**) 0.5% SiO_2_/Co_2.4_Ni_0.6_O_4_. The dotted lines correspond to the theoretical values and the solid ones to the experimental ones.

**Figure 11:**
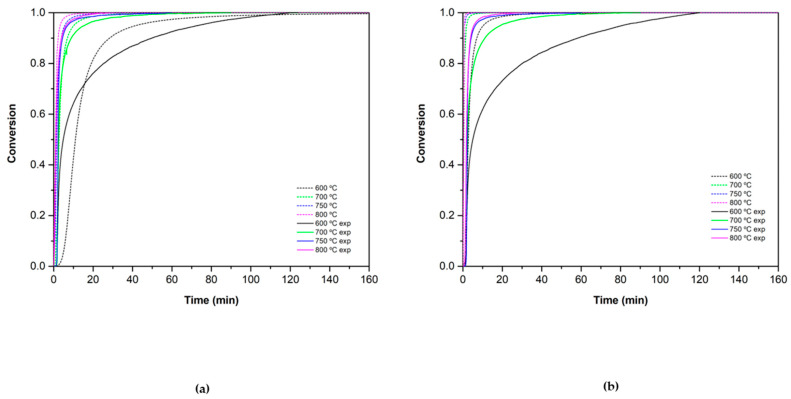
Oxidation conversion validation for (**a**) Co_2.4_Ni_0.6_O_4_ and (**b**) 0.5% SiO_2_/ Co_2.4_Ni_0.6_O_4_. The dotted lines correspond to the theoretical values and the solid ones to the experimental ones.

The concordance of the experimental results and the theoretical values were assessed by using the OCTAVE software. The theoretical equations (Equations (15), (16), (19) and (20)) were implemented in the program to obtain the predicted behavior of the materials and were compared with the experimental data obtained in the TGA. Figure 10 and Figure 11 show this comparison for the reduction and oxidation reactions, respectively.

In the case of Co_2.4_Ni_0.6_O_4_ (Figure 10A), the theoretical values obtained fit well with the experimental data until conversion ratios of 0.8, where a drop in the speed in the model is observed. This may be because, at conversion ratios above 0.5, the nucleation and growth model governs the course of the reaction, so the SB equation does not fully define the trend of the experimental data. In the case of the 0.5% SiO_2_/Co_2.4_Ni_0.6_O_4_ (Figure 10B), the experimental data fit well with the theoretical one as diffusion mechanisms govern the whole reaction extent range. For both cases, the calculated model does not fit completely with the experimental data using a heating ramp of 5 °C/min. In this case, the heating ramp could be too slow to observe the normal course of the reaction.

The oxidation reaction validation is shown in Figure 11. Experimental and theoretical data of Co_2.4_Ni_0.6_O_4_ show a good concordance in all the reaction extent. In the case of the added 0.5% SiO_2_/Co_2.4_Ni_0.6_O_4_, the theoretical trend corresponds well until conversion values of 0.8. The conversion ranges from 0.8 to 1 are not fully adjusted with the SB equation. In both materials, different behavior is observed between theoretical and experimental results at a temperature of 600 °C. As was the case with the reduction step, the isotherm at 600 °C may not have a fully adequate temperature for the normal course of the reaction without interference or limitations from the process temperature.

## 4. Conclusions

In this work, the development of a kinetic model of Co_2.4_Ni_0.6_O_4_ and of 0.5% SiO_2_/Co_2.4_Ni_0.6_O_4_ materials for thermochemical energy storage applications was firstly reported. The results obtained for these materials were compared with the data from the pure cobalt oxide available from previous research works.

In the reduction reaction, the activation energy of the two materials (450 kJ/kg for Co_2.4_Ni_0.6_O_4_ and 449 kJ/kg for 0.5% SiO_2_/Co_2.4_Ni_0.6_O_4_) is in the range of the pure Co_3_O_4_ one (158–960 kJ/kg), so neither the nickel substitution in the cobalt oxide structure nor the addition of SiO_2_ particles affects this parameter.

The theoretical models *f*(*α*) fitting with the reduction experimental data are different nucleation and growth mechanisms in the case of the Co_2.4_Ni_0.6_O_4_ and different diffusion models in the case of 0.5% SiO_2_/Co_2.4_Ni_0.6_O_4_.

In the oxidation reaction, the activation energy of the two materials (99 kJ/kg for Co_2.4_Ni_0.6_O_4_ and 123 kJ/kg for 0.5% SiO_2_/Co_2.4_Ni_0.6_O_4_) is in agreement with the values published in regard to other mixed oxides (165 kJ/kg for 5% Al_2_O_3_/Co_3_O_4_) but higher than the value reported in the bibliography for Co_3_O_4_ (~60 kJ/kg).

The behavior of the two materials fits with the same theoretical models *f*(*α*) describing nucleation and growth mechanisms when the conversion ratios are higher than 0.5. The SB equation was used to obtain the model of the whole experiment.

The developed model confirms a good agreement between the experimental data and the theoretical ones in most of the measurement conditions.

## Figures and Tables

**Table 1:** Kinetic models (*f*(*α*)) for the most representative gas–solid reactions.

Reaction Model	Name	Mechanism	*f*(*α*)
Reaction order models	F1	Random nucleation followed by an instantaneous growth of nuclei	1−*α*
	F2		(1−*α*)^2^
	F3		(1−*α*)^3^
Power law	P2	Random nucleation and growth of nuclei through different nucleation and nucleus growth models	2*α*^1/2^
	P3		3*α*^2/3^
	P4		4*α*^3/4^
Avrami–Erofeev	A2		2(1−*α*)[−ln(1−*α*)]^1/2^
	A3		3(1−*α*)[−ln(1−*α*)]^2/3^
	A4		4(1−*α*)[−ln(1−*α*)]^3/4^
Contracting area	R2	Phase boundary-controlled reaction	2(1−*α*)^1/2^
Contracting volume	R3		3(1−*α*)^2/3^
Diffusion	D1	Based on the penetration of reactant molecules through a layer of product	½*α*−1
	D2		[−ln(1−*α*)]^−1^
	D3		3/2(1−*α*)^2/3^[1−(1−*α*)1/3]^−1^
	D4		3/2[(1−*α*)−1/3−1]

**Table 2:** Elemental composition of 0.5% SiO_2_/Co_2.4_Ni_0.6_O_4_ obtained in EDX.

Element	Wt%	At%
SiK	0.00	0.00
NiK	78.55	78.49
CoK	21.45	21.51

**Table 3:** Kinetic triplet values describing the reduction reaction of the Co_2.4_Ni_0.6_O_4_ and 0.5% SiO_2_/Co_2.4_Ni_0.6_O_4_ materials and activation energy values of pure Co_3_O_4_ and 5% Al_2_O_3_/Co_3_O_4_.

Material	*Ea* (kJ/mol)	*f*(*α*)			*A* (min^−1^)
		*m*	*n*	*p*	
Co_2.4_Ni_0.6_O_4_	450 ± 47	2	1.7	−2.1	9.65·10^19^
0.5% SiO_2_/Co_2.4_Ni_0.6_O_4_	449 ± 32	0.81	1.45	−0.87	6.37·10^19^
Co_3_O_4_ [25]	247	-	-	-	-
Co_3_O_4_ [26]	960	-	-	-	-
5% Al_2_O_3_/Co_3_O_4_ [29]	238	-	-	-	-
5% Al_2_O_3_/Co_3_O_4_ [26]	731	-	-	-	-

**Table 4:** Kinetic triplet values describing the oxidation reaction of the Co_2.4_Ni_0.6_O_4_ and 0.5% SiO_2_/Co_2.4_Ni_0.6_O_4_ materials and activation energy values of pure Co_3_O_4_ and 5% Al_2_O_3_/Co_3_O_4_.

Material	*Ea* (kJ/mol)	*f*(*α*)			*A* (min^−1^)
		*m*	*n*	*p*	
Co_2.4_Ni_0.6_O_4_	100 ± 22	2.8	1	−2.01	7·10^3^
0.5% SiO_2_/Co_2.4_Ni_0.6_O_4_	134 ± 36	2.8	1	−2.01	7.81·10^5^
Co_3_O_4_ [25]	58 ± 0.26	-	-	-	-
Co_3_O_4_ [41]	60	-	-	-	-
5% Al_2_O_3_/Co_3_O_4_ [26]	165	-	-	-	-
